# Supplementary material for: FastqPuri: high-performance preprocessing of RNA-seq data
Source: BMC Bioinformatics. 2019 May 3;20:226. doi: 10.1186/s12859-019-2799-0 (PMC6500068; doi:10.1186/s12859-019-2799-0)
Supplement: Supplementary file 2 — Archive of FastqPuri. Archive containing all files needed to install and run FastqPuri v1.0.6. Date stamp March 22, 2019. (GZ 47,819 kb) [file 12859_2019_2799_MOESM2_ESM.gz › FastqPuri-1.0.6/html/functions_vars.html]

FastqPuri: Class Members - Variables


FastqPuri


### - a -

- ACGT\_pos
  : statsinfo
- ACGT\_tile
  : statsinfo
- ad
  : \_iparam\_trimFilter
- ad1
  : \_ds\_adap
- ad2
  : \_ds\_adap
- ad2\_fa
  : \_adapter
- ad\_fa
  : \_adapter

### - b -

- bfsizeBits
  : \_bfilter
  , \_iparam\_makeBloom
- bfsizeBytes
  : \_bfilter

### - c -

- children
  : \_node
- compact
  : \_bfkmer

### - d -

- discarded
  : \_stats\_TF
  , \_stats\_TFDS

### - e -

- entry
  : \_fa\_data
- entrylen
  : \_fa\_data
- extended
  : \_fq\_read

### - f -

- falsePosRate
  : \_bfilter
  , \_iparam\_makeBloom
- filter
  : \_bfilter
  , \_iparam\_Qreport
- filterfile
  : \_iparam\_makeBloom
- filters
  : \_stats\_TF
  , \_stats\_TFDS

### - g -

- globleft
  : \_iparam\_trimFilter
- globright
  : \_iparam\_trimFilter
- good
  : \_stats\_TF
  , \_stats\_TFDS

### - h -

- halfsizeBytes
  : \_bfkmer
- hangingBases
  : \_bfkmer
- hashNum
  : \_bfilter
  , \_bfkmer
  , \_iparam\_makeBloom
- hashValues
  : \_bfkmer
- hasOverhead
  : \_bfkmer

### - i -

- Ifa
  : \_iparam\_trimFilter
- Ifq
  : \_iparam\_trimFilter
- Ifq2
  : \_iparam\_trimFilter
- Iidx
  : \_iparam\_trimFilter
- Iinfo
  : \_iparam\_trimFilter
- inputfasta
  : \_iparam\_makeBloom
  , \_iparam\_makeTree
- inputfile
  : \_iparam\_Qreport
- inputfolder
  : \_iparam\_Sreport
- is\_adapter
  : \_iparam\_trimFilter
- is\_fa
  : \_iparam\_trimFilter
- is\_idx
  : \_iparam\_trimFilter

### - k -

- kmersize
  : \_bfilter
  , \_bfkmer
  , \_iparam\_makeBloom
  , \_iparam\_trimFilter
- kmersizeBytes
  : \_bfilter
  , \_bfkmer

### - l -

- L
  : \_ad\_seq
  , \_fq\_read
  , \_iparam\_makeTree
  , \_iparam\_trimFilter
  , \_tree
- L1
  : \_ds\_adap
- L2
  : \_ds\_adap
- L\_ad
  : \_fq\_read
- L\_ext
  : \_fq\_read
- L\_pack
  : \_fq\_read
- L\_packsh
  : \_fq\_read
- lane\_tags
  : statsinfo
- Lhalf
  : \_fq\_read
- line1
  : \_fq\_read
- line2
  : \_fq\_read
- line3
  : \_fq\_read
- line4
  : \_fq\_read
- linelen
  : \_fa\_data
- lowQ\_ACGT\_tile
  : statsinfo
- Lpack
  : \_ad\_seq
- Lpack\_sh
  : \_ad\_seq

### - m -

- method
  : \_iparam\_trimFilter
- minL
  : \_iparam\_trimFilter
- minQ
  : \_iparam\_Qreport
  , \_iparam\_trimFilter
  , statsinfo
- mismatches
  : \_adapter

### - n -

- N
  : \_fa\_entry
  , \_split
- Nad
  : \_adapter
- nelem
  : \_bfilter
  , \_iparam\_makeBloom
- nentries
  : \_fa\_data
- nlines
  : \_fa\_data
- nlowQ
  : \_iparam\_trimFilter
- nnodes
  : \_tree
- nQ
  : \_iparam\_Qreport
  , statsinfo
- nreads
  : \_stats\_TF
  , \_stats\_TFDS
  , statsinfo
- ntiles
  : \_iparam\_Qreport
  , statsinfo

### - o -

- one\_read\_len
  : \_iparam\_Qreport
- Oprefix
  : \_iparam\_trimFilter
- outputfile
  : \_iparam\_makeTree
  , \_iparam\_Sreport
- outputfilebin
  : \_iparam\_Qreport
- outputfilehtml
  : \_iparam\_Qreport
- outputfileinfo
  : \_iparam\_Qreport

### - p -

- pack
  : \_ad\_seq
  , \_fq\_read
- pack\_sh
  : \_ad\_seq
- packsh
  : \_fq\_read
- paramfile
  : \_iparam\_makeBloom
- percent
  : \_iparam\_trimFilter
- pool\_2D
  : \_tree
- pool\_available
  : \_tree
- pool\_count
  : \_tree
- ptr\_bfkmer
  : \_iparam\_trimFilter

### - q -

- QPosTile\_table
  : statsinfo
- qual\_tags
  : statsinfo

### - r -

- read\_len
  : \_iparam\_Qreport
  , statsinfo
- reads\_MlowQ
  : statsinfo
- reads\_wN
  : statsinfo
- Rmd\_file
  : \_iparam\_Sreport

### - s -

- s
  : \_split
- score
  : \_iparam\_trimFilter
- seq
  : \_ad\_seq
  , \_fa\_entry
- start
  : \_fq\_read
- sz\_ACGT\_pos
  : statsinfo
- sz\_ACGT\_tile
  : statsinfo
- sz\_lowQ\_ACGT\_tile
  : statsinfo
- sz\_QPosTile\_table
  : statsinfo
- sz\_reads\_MlowQ
  : statsinfo

### - t -

- threshold
  : \_adapter
- tile\_pos
  : statsinfo
- tile\_tags
  : statsinfo
- trimmed
  : \_stats\_TF
- trimmed1
  : \_stats\_TFDS
- trimmed2
  : \_stats\_TFDS
- trimN
  : \_iparam\_trimFilter
- trimQ
  : \_iparam\_trimFilter

### - u -

- uncompress
  : \_iparam\_trimFilter


---

Generated on Mon Mar 19 2018 23:42:01 for FastqPuri by  

 1.8.14
